# Lovastatin Protects against Experimental Plague in Mice

**DOI:** 10.1371/journal.pone.0010928

**Published:** 2010-06-02

**Authors:** Saravanan Ayyadurai, Hubert Lepidi, Claude Nappez, Didier Raoult, Michel Drancourt

**Affiliations:** Unité de Recherche sur les Maladies Infectieuses et Tropicales Emergentes: UMR CNRS 6236- IRD 198, Faculté de Médecine, IFR48, Université de la Méditerranée, Marseille, France; Columbia University, United States of America

## Abstract

**Background:**

Plague is an ectoparasite-borne deadly infection caused by *Yersinia pestis*, a bacterium classified among the group A bioterrorism agents. Thousands of deaths are reported every year in some African countries. Tetracyclines and cotrimoxazole are used in the secondary prophylaxis of plague in the case of potential exposure to *Y. pestis*, but cotrimoxazole-resistant isolates have been reported. There is a need for additional prophylactic measures. We aimed to study the effectiveness of lovastatin, a cholesterol-lowering drug known to alleviate the symptoms of sepsis, for plague prophylaxis in an experimental model.

**Methodology:**

Lovastatin dissolved in Endolipide was intraperitoneally administered to mice (20 mg/kg) every day for 6 days prior to a *Y. pestis* Orientalis biotype challenge. Non-challenged, lovastatin-treated and challenged, untreated mice were also used as control groups in the study. Body weight, physical behavior and death were recorded both prior to infection and for 10 days post-infection. Samples of the blood, lungs and spleen were collected from dead mice for direct microbiological examination, histopathology and culture. The potential antibiotic effect of lovastatin was tested on blood agar plates.

**Conclusions/Significance:**

Lovastatin had no in-vitro antibiotic effect against *Y. pestis*. The difference in the mortality between control mice (11/15; 73.5%) and lovastatin-treated mice (3/15; 20%) was significant (*P*<0.004; Mantel-Haenszel test). Dead mice exhibited *Y. pestis* septicemia and inflammatory destruction of lung and spleen tissues not seen in lovastatin-treated surviving mice. These data suggest that lovastatin may help prevent the deadly effects of plague. Field observations are warranted to assess the role of lovastatin in the prophylaxis of human plague.

## Introduction

*Yersinia pestis* is a Gram-negative bacillus belonging to the family *Enterobacteriaceae*. It is responsible for deadly bubonic, pneumonic and septicemic plagues [1], [2] and is classified as a group A bioterrorism agent (CDC, Atlanta, GA). Africa remains the continent reporting the highest number of human plague cases per year, with the Democratic Republic of the Congo declaring 1,700 to 2,000 cases and Madagascar declaring 500 to 600 cases to the World Health Organization each year [http://www.who.int/csr/don/archive/disease/plague/en/]. Experimental animal models have indicated that, after inoculation, *Y. pestis* rapidly escapes containment in the lymph node, spreads systemically through the blood and produces fatal sepsis [2]. Sepsis occurs when the immune system of the host responds to a localized infection at a systemic level and thereby causes tissue damage and organ dysfunction [3]. Clinical observations indicated that statins, which are competitive inhibitors of hydroxymethylglutaryl-coenzyme A (HMG-CoA) [4], [5], could prevent infections and reduced mortality during severe sepsis [6]. Recent animal data has confirmed that the administration of statins before a sepsis-inducing insult reduced morbidity and improved survival [7], [8]. No data have been published regarding the potential role of statins in the prevention of mortality during plague. We therefore tested whether lovastatin, a statin obtained from fungal fermentation, could significantly reduce the mortality associated with plague in an experimental mouse model.

## Materials and Methods

### Ethics Statement

All studies were reviewed and approved by the Institutional Animal Care and Use Committee at the Medical Faculty of Marseille.

### Bacterial strain and in vitro testing of lovastatin susceptibility

*Y. pestis* strain 6/69M biotype Orientalis, a virulent isolate originally from Madagascar (kindly provided by Prof. Michel Simonet, Institut Pasteur, Lille, France) was grown on 5% sheep-blood agar (BioMérieux, Marcy l'Etoile, France) at 28°C under a 5% CO_2_ atmosphere for 2 days before use. The in-vitro antibiotic activity of lovastatin (Sigma Aldrich, Saint-Quentin Fallavier, France) was checked by pipetting 100 µl of a 4 mg/ml lovastatin/Endolipide (B. Braun Melsungen AG, France) solution into two 0.5-cm^3^ wells of a 5% sheep-blood agar plate (BioMérieux) inoculated with *Y. pestis* 6/69M Orientalis. Plates were then incubated at 30°C for two days to check for any inhibition zone around the lovastatin wells. The experiment was performed in triplicate.

### Animals and experimental protocol

A total of 45 six- to eight-week-old (16–18 g) female BALB/c mice were purchased from Charles River Laboratories (Saint-Aubin-les-Elbeuf, France). Animals were housed in BSL3 containment for 3–5 days before treatment. As a preliminary control, 3 mice were injected intraperitoneally with 100 µL Endolipide alone; these mice remained alive and symptom-free for 7 days. For treatment, one group of 15 animals was injected intraperitoneally with Endolipide every 24 h for 6 days (group A, *Y. pestis* 6/69M control group); a second group of 15 animals was injected intraperitoneally with 20 mg/kg lovastatin solubilized in Endolipide every 24 h for 6 days (group B, prophylaxis group); a third group of 15 mice were injected intraperitoneally with 20 mg/kg lovastatin solubilized in Endolipide (group C, lovastatin control group) and were also maintained throughout the experiment. Groups A and B were challenged with *Y. pestis* 6/69M 6 hours after the last lovastatin injection by intraperitoneal injection of 100 µl of a 10^8^ cfu/ml suspension of *Y. pestis* 6/69M in PBS. Inoculated animals were observed for the development of signs of lethal plague disease, including loss of body weight, altered physical behavior and death, for a period of 10 days. After the observation period, the remaining animals were humanely euthanized by CO_2_ asphyxiation, a method approved by the Panel on Euthanasia of the American Veterinary Medical Association. Euthanized animals were necropsied and blood was drawn by cardiac puncture.

### Detection of *Y. pestis*

Blood samples collected by intracardiac puncture 24 to 48 hours p.i. from dead mice were spotted on a slide, spread using another slide and air dried at room temperature. Slides were fixed in 100% methanol for 10 min and stained with freshly prepared acridine orange in the dark for 5 min [9]. Slides were then rinsed with tap water, air dried at room temperature, overlaid with DAPI (4′, 6′-diamidino-2-phenylindole) ready-to-use solution (Molecular Probes, Montluçon, France) and examined using a Leica DM 2500 upright fluorescence microscope at 100× magnification with a FITC-rhodamine double band filter. A second blood drop was deposited on a slide with a pen nib for indirect immunofluorescence detection using an anti-*Y. pestis* rabbit polyclonal antibody and FITC-conjugated goat anti-rabbit IgG (Immunotech, Marseille, France) diluted at 1∶400 in PBS containing 3% non-fat dry milk and 0.2% Evans blue (BioMérieux, Marcy l'Etoile, France). Slides were washed, air dried and mounted with Fluoroprep (BioMérieux) and then examined under a Olympus BX-51 epifluorescence microscope at 100× magnification. A third drop of blood was inoculated on a blood agar plate (BioMérieux) and incubated at 28°C in a 5% CO_2_ atmosphere for 2 days. To identify the colonies as *Y. pestis*, DNA was extracted from the colonies using the QIAGEN-QIAamp DNA Mini Kit according to the manufacturer protocol (QIAGEN S. A. Courtaboeuf, France) for PCR amplification of the plasminogen-activator gene *pla* [10]. PCR was carried out in the Applied Biosystems 2720 thermal cycler (MJ Research, Waltham, Mass). For the reaction, 1 µg/µl of the DNA preparation was amplified in a 50-µl reaction mixture containing 10 mM Tris-HCl (pH 8.4), 50 mM KCl, 1.5 mM MgCl_2_, 0.2 mM dNTPs (Invitrogen, Gaithersburg, Md.), 0.1 mM primer (Eurogentec S A, Seraing, Belgium) and 1 U of *Taq* DNA polymerase (Invitrogen) at a final volume of 50 µl. *pla* gene amplification was performed using an initial 5-min denaturation step at 95°C and 39 cycles of 1-min denaturation at 94°C, 30-s annealing at 60°C and 1-min extension at 74°C followed by a final 15-min extension at 75°C. Sterilized water was used as a negative control in each PCR assay and no positive control was used. PCR products were purified using the multi-screen PCR filter plate (Millipore, Saint-Quentin en Yvelines, France). Sequencing reactions were performed using the d-Rhodamine Terminator Cycle Sequencing Ready Reaction kit with Amplitaq polymerase FS (Perkin-Elmer, Coignières, France) and the sequencing products were resolved using an ABI 3100 automated sequencer (Perkin-Elmer). Sequence analysis was performed using the ABI prism DNA sequencing analysis software package version 3.0 (Perkin-Elmer) on a Power Macintosh 7200/120. The GenBank database was referenced via the internet with BLAST software from the National Center for Biotechnology Information homepage (http://www.ncbi.nlm.nih.gov/BLAST/) and the sequence results were compared.

### Pathological analyses

After the mice died, the lungs and the spleen were removed, fixed with 4% buffered formalin and embedded in paraffin. Serial sections (3 µm) of tissue specimens were obtained for routine hematoxylin-eosin-saffron (HES) and Giemsa staining.

### Mantel-Haenszel chi-square test

The Mantel-Haenszel test [11] was used to compare the outcomes of the groups. The numerical values of live and dead mice were entered into the table, and the statistical test was performed. A p value <0.05 was considered significant.

## Results

### In vitro susceptibility of *Y. pestis* to lovastatin

Sequencing the *pla* gene (100% sequence identity with GenBank accession number AL109969.1) confirmed identification of this strain at the time of the experiment. In vitro testing indicated that *Y. pestis* grew when in contact with lovastatin-containing wells.

### Mouse clinical data

**Figure 1:**
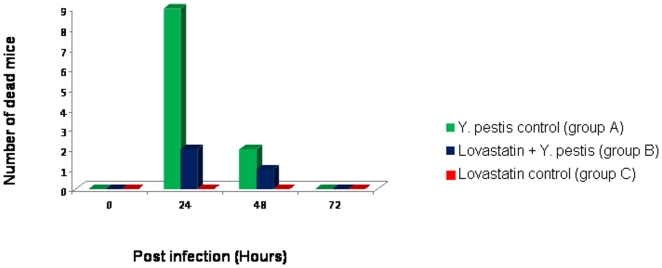
Histogram of mortality in groups of 15 mice challenged with *Y. pestis* and injected with PBS (group A) or lovastatin (group B); or challenged with lovastatin only (group C).

A total of 11/15 (73.5%) control group A mice inoculated with *Y. pestis* died within 48 hours post injection (p.i.), whereas 3/15 (20%) of the lovastatin group B mice died over a 10-day observation period (*P*<0.004) (Figure 1). In both groups, inoculated mice exhibited hair loss and a decreased appetite, which was not observed in mice from control group C. In the *Y. pestis*-infected control group A, weight decreased from 15. 8 g±0.4 g at the time of inoculation to 15.0 g±0.4 g at 24 h p.i. and 14.6 g±0.4 g at 48 h p.i., whereas the lovastatin control group C mice demonstrated an increase in weight from 16.1±0.4 g at the time of inoculation to 19.1±0.4 g at the end of the 10-day observation period. The weight of the lovastatin-treated group B mice increased from 16.2±0.4 g at the time of lovastatin treatment to 17.1±0.4 g during the of 6-day lovastatin treatment period and then decreased 0.9±0.2 g after 48 h after the *Y. pestis* challenge, gradually increasing to 18.9±0.4 g by the end of the 10-day observation period.

### Microbiological and pathology data

**Figure 2:**
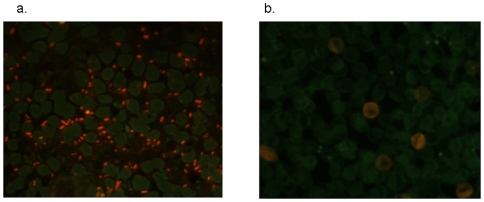
Acridine orange staining of blood from dead group B mice (a; original magnification, 100×) showing *Y. pestis* organisms. No organisms were found in the blood of the lovastatin-treated mice that survived the *Y. pestis* challenge (b; original magnification, 100×).

**Figure 3:**
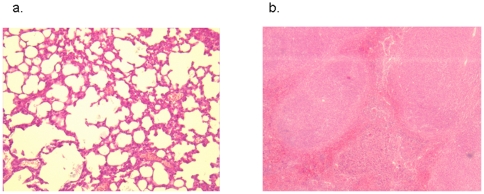
HES staining of lung tissue (a; original magnification, 100×) and spleen tissue (b; original magnification ×50) from group B mice after lovastatin prophylaxis and *Y. pestis* challenge. No pathological changes were observed; particularly, no inflammatory infiltrates or necrotic areas were detected.

Microscopy detected acridine orange stained *Y. pestis* in the blood (Figure 2) and lung tissue of 11/15 (73.5%) group A mice, as well as in 3/15 (20%) group B mice, but not in group C mice. In mice from groups A and B, culturing the blood and lung tissue resulted in colonies identified as *Y. pestis* by *pla* sequence amplification, whereas cultures of blood and lung tissue from group C mice remained sterile. With group A mice, further microscopic examination demonstrated that the lungs had lost their recognizable architecture, although some of the larger bronchi were still visible. Along with extensive hemorrhages with large foci of necrosis, large numbers of neutrophils were packed within the remaining pulmonary interstitium. The spleen showed extensive necrosis and neutrophilic infiltration. Giemsa-stained bacteria were observed in great numbers in the pulmonary interstitial vessels and connective tissues but not in the alveolar spaces or in the lumen of bronchi. Large numbers of extracellular bacteria were also seen in the spleen, mainly in the red pulp. In contrast, group B mice that survived *Y. pestis* challenge presented no damage in the lungs and spleen. The pulmonary interstitium and the splenic pulp showed no inflammatory infiltrates or necrosis,and there were no bacteria detected by Giemsa staining (Figure 3).

## Discussion

In the conditions of this experimental study, lovastatin significantly protected animals against a deadly challenge with the plague agent *Y. pestis*. In this study, the intraperitoneal route was used for producing a rapidly fatal, disseminated infection in mice as previously reported [12]. In this study, lovastatin was administrated by the parenteral route in order to ensure the dosage. Because lovastatin is not readily soluble in aqueous solution, we dissolved it in Endolipide, a lipidic solution also licensed for parenteral administration in humans. We did ensure that intraperitoneal Endolipide had no deleterious effect on mice and did not protect mice from deadly plague.

After *Y. pestis* challenge, both control and lovastatin-treated mice exhibited symptoms of plague, including loss of appetite and decreased weight, but most of the lovastatin-treated mice recovered 48 hours after *Y. pestis* challenge, whereas three-quarters of the untreated animals died. Dead mice were all bacteremic with characteristic lesions in the lungs and spleen, whereas the surviving, lovastatin-treated mice had no bacteremia and no pathological lesions in their organs in day-10 after challenge; as transient bacteremia was not surveyed in this study, it was not possible to assess the role of lovastatin in preventing dissemination of *Y. pestis*. Indeed, we did not observe any direct antibacterial effect of lovastatin-Endolipide using in vitro tests, which is in agreement with the fact that the *Y. pestis* genome does not encode HMG-CoA reductase, the molecular target for lovastatin [12]–[16]. These data therefore suggest that lovastatin had no direct effect on inoculated *Y. pestis* organisms but, rather, exhibited anti-*Yersinia* activity after *Y. pestis* organisms came into contact with host cells. Accordingly, a previous study showed that lovastatin had no effect on the growth of *Salmonella enterica* Typhimurium in broth but caused a significant reduction in its intracellular growth in RAW 264.7 murine macrophages [8]. Also, lovastatin was shown to lower the growth of the strictly intracellular *Coxiella burnetii* and *Rickettsia conorii* when co-cultivated with the murine fibroblast L929 cell line [17], [18].

The data reported herein are of interest regarding the prophylactic treatment of plague. Primary prophylaxis aims to prevent contact with *Y. pestis* by relying on barrier measures, including avoiding areas with known epizootic plague, avoiding animals appearing to be sick or dead, avoiding exposure to fleas from diseased rats by avoiding places that are infested with rats or where large numbers of rats have reportedly died, using protective clothes and repellents to avoid exposure to ectoparasites when outdoors and applying insect repellent containing DEET to legs and ankles. Additional preventive methods include applying repellents and insecticides to clothes and outer bedding, using gloves for the manipulation of carcasses and cooking meat on an open-flame grill or on a clam-shell type electric grill [19]. In the case of potential contact with the plague agent, secondary prophylaxis relies on the rapid administration of selected antibiotics comprised of tetracyclines and cotrimoxazole [20]. *Y. pestis* organisms are susceptible to most antibiotics that are active against gram-negative organisms, except for imipenem, rifampin and macrolides [21]–[23], but a clinical *Y. pestis* isolate from Madagascar has been reported to be resistant to ampicillin, chloramphenicol, kanamycin, streptomycin, spectinomycin, sulfonamides, tetracycline and minocycline [24]. A second clinical isolate resistant to streptomycin has been further characterized in Madagascar [25]. In both isolates, resistance genes were carried by a transferable plasmid [25]. Based on experimental data gathered in fleas, it has been hypothesized that horizontal gene transfer in the flea may have been the source of such antibiotic-resistant *Y. pestis* strains [26]. Such resistance includes resistance to antibiotics used in the secondary prophylaxis of human plague [20]. Also, *Y. pestis* isolates resistant to fluoroquinolones have been easily selected by experimental growth in the presence of the targeted antibiotic [20], [27]–[29], and the selection of streptomycin-resistant strains has been observed in an experimental model of pneumonic plague treated with streptomycin [30]. These data indicate both a potential for populations to be exposed to a natural or engineered antimicrobial-resistant *Y. pestis* strain and the probable failure of current prophylaxis schemes based on antibiotics [31].

With respect to this eventuality, field observations measuring plague incidence and severity would be warranted to assess the potential of lovastatin for lowering plague incidence and severity in individuals receiving lovastatin as part of anticholesterol treatment, and exposed to plague endemic countries.
